# Favorable effects of burosumab on tumor-induced osteomalacia caused by an undetectable tumor: A case report

**DOI:** 10.1097/MD.0000000000027895

**Published:** 2021-11-19

**Authors:** Yuki Oe, Hiraku Kameda, Hiroshi Nomoto, Keita Sakamoto, Takeshi Soyama, Kyu Yong Cho, Akinobu Nakamura, Koji Iwasaki, Daisuke Abo, Kohsuke Kudo, Hideaki Miyoshi, Tatsuya Atsumi

**Affiliations:** a Department of Rheumatology, Endocrinology and Nephrology, Faculty of Medicine and Graduate School of Medicine, Hokkaido University, Sapporo, Japan; b Department of Diagnostic and Interventional Radiology, Hokkaido University Hospital, Sapporo, Japan; c Department of Orthopedic Surgery, Faculty of Medicine and Graduate School of Medicine, Hokkaido University, Sapporo, Japan; d Department of Diagnostic Imaging, Faculty of Medicine and Graduate School of Medicine, Hokkaido University, Sapporo, Japan; e Global Center for Biomedical Science and Engineering, Faculty of Medicine, Hokkaido University, Sapporo, Japan; f Division of Diabetes and Obesity, Faculty of Medicine and Graduate School of Medicine, Hokkaido University, Sapporo, Japan.

**Keywords:** burosumab, hypophosphatemia, osteocalcin, treatment, tumor-induced osteomalacia, undetectable tumor

## Abstract

**Rationale:**

Tumor-induced osteomalacia (TIO) is curable by tumor resection, but detection of the tumor can be challenging. Overproduction of fibroblast growth factor 23 (FGF23) by the tumor causes hypophosphatemia and consequently induces inappropriate bone turnover. Conventionally oral phosphate supplementation was the only treatment for TIO, but had risks of hypercalciuria and nephrocalcinosis. Burosumab, a human monoclonal anti-FGF23 antibody, was recently post-marketed in Japan against for FGF23-related hypophosphatemia. Herein, we present a case of TIO with undetectable tumor that was successfully treated with burosumab.

**Patient concerns:**

A 47-year-old woman was forced to use a wheelchair because of pain in both feet.

**Diagnosis:**

Laboratory findings showed hypophosphatemia, elevated bone markers, and high serum FGF23 without renal tubular defects. Imaging studies revealed bone atrophy in the feet, decreased bone density, and multiple pseudofractures in the talar, sacral, and L5 vertebral regions. After excluding drug-induced and hereditary osteomalacia, we diagnosed her as TIO.

**Interventions:**

Comprehensive imaging studies and stepwise venous sampling failed to localize the tumor, and we started to administer subcutaneous burosumab.

**Outcomes:**

After administration of burosumab, her serum phosphate was normalized without phosphate supplementation within 2 months. Improvement of pseudofractures, relief of pain evaluated by a visual analog scale, and normalization of bone biomarkers were observed. The patient was able to stand by herself after 6 months administration of burosumab.

**Lessons:**

This is the first report in clinical practice to demonstrate favorable effects of burosumab, including not only normalization of serum phosphate but also improvements of pseudofractures and subjective pain, in a patient with TIO and undetectable tumor.

## 1 Introduction

Fibroblast growth factor 23 (FGF23) decreases serum phosphate by inhibiting proximal tubular phosphate reabsorption and reduces intestinal phosphate absorption by lowering serum 1,25-dihydroxyvitamin D, thereby regulating phosphate metabolism.^[1]^

Many of the typical FGF23-related hypophosphatemic diseases are hereditary, including X-linked hypophosphatemic rickets and autosomal dominant hypophosphatemic rickets.^[2]^ A few cases with adult-onset hypophosphatemia in patients on saccharated ferric oxide have been documented as well.^[3]^

Tumor-induced osteomalacia (TIO) is a rare paraneoplastic syndrome caused by FGF23 overproduction by the tumor tissues.^[1]^ Resection of the responsible tumor, which is usually located in bone or soft tissue, is a curative treatment for TIO. When the responsible tumor is undetectable, phosphate supplementation and active vitamin D administration are recommended for the treatment against TIO.^[4]^ However, medical adherence to oral phosphate supplementation is sometimes poor because of the multiple daily administrations required and failure to maintain phosphate levels. The complications associated with this medical therapy, including hypercalciuria and nephrocalcinosis, are also difficult to prevent.^[5]^

In December 2019, burosumab, a human monoclonal anti-FGF23 antibody, was approved in Japan as a therapeutic agent for FGF23-related hypophosphatemia. Although subcutaneous burosumab administration has already been established as an effective treatment for X-linked hypophosphatemic rickets, its potential as a treatment for TIO has not been determined and only a few case reports and clinical trials have been presented.^[6–10]^ Herein, we describe a case of TIO caused by an undetectable tumor that was effectively treated with burosumab.

## 2 Case presentation

A 47-year-old female developed pain and edema of the feet. The cause of her pain was undetermined, and subsequently, she developed marked bone atrophy in the feet and was referred to our hospital. She had no problems for walking until the symptom onset, but she could not stand. She had a height of 158.2 cm and a body weight 48.6 kg, giving a body mass index 19.5 kg/m^2^. She had a history of smoking 20 cigarettes a day for 20 years and only occasionally drank alcohol. There were no reports of relevant family history and her menstrual history was normal. She was not receiving any medication at the time of her visit, including saccharated iron oxide nor iron polymaltose. Physical examination did not show abnormal findings except for pain during dorsiflexion at both talocrural joints.

Initial laboratory findings at our facility showed serum phosphate of 1.9 mg/dL (reference range: 2.7–4.6 mg/dL), 25-hydroxyvitamin D 20.0 ng/mL (20.0–100.0 ng/mL), 1,25-dihydroxyvitamin D 12.9 pg/mL (20.0–60.0 pg/mL), calcium 9.3 mg/dL (8.8–10.1 mg/dL), intact parathyroid hormone (PTH) 75.0 pg/mL (8.7–79.6 pg/mL), bone alkaline phosphatase 36.7 μg/L (2.9–14.5 μg/L), type 1 collagen cross-linked N-terminal telopeptide (NTx) 31.5 nmol BCE/L (7.5–16.5 nmol BCE/L), and tartrate-resistant acid phosphatase 5b (TRACP-5b) 1330.0 mU/dL (120.0–420.0 mU/dL). Other laboratory findings including biochemistry and endocrinology were normal (Table 1). Urinary fractional excretion of phosphate was inappropriately normal at 16.7% (15.0%–20.0%) given her hypophosphatemia and renal tubular reabsorption of phosphate was low at 1.6 mg/dL (2.3–4.3 mg/dL). Serum FGF23 (Kainos assay, ELISA) was remarkably elevated at 630.0 pg/mL (16.0–69.0 pg/mL). Therefore, she was diagnosed as hypophosphatemic osteomalacia was diagnosed.

**Table 1:** Laboratory findings on admission.

<Urine testing>			<CBC>			BUN	9.0	mg/dL	<Endocrinology>		
pH	5.5		WBC	5100	/μL	Cre	0.40	mg/dL	ACTH	13.68	pg/mL
Protein	+/-		RBC	4.19 × 10^6^	/μL	eGFR	127.9	mL/min/1.73m^3^	Cortisol	3.2	μg/dL
Glucose	-		Hb	12.6	g/dL	Na	141	mEq/L	GH	0.83	ng/mL
Ketone	-		Ht	38.5	%	K	4.1	mEq/L	IGF-1	117	ng/mL
Blood	+/-		Plt	24.6 × 10^4^	/μL	Cl	106	mEq/L	LH	4.0	mIU/mL
<Urine Serology>			<Biochemistry>			Ca	9.3	mg/dL	FSH	4.5	mIU/mL
U-UN	872	mg/dL	T-bil	0.9	mg/dL	P	1.9	mg/dL	Estradiol	72.0	pg/mL
U-Cr	121	mg/dL	AST	18	U/L	Mg	1.9	mg/dL	PRL	6.6	ng/mL
U-UA	54.6	mg/dL	ALT	14	U/L	CRP	0.1	mg/dL	TSH	1.36	mIU/mL
U-Na	91	mEq/L	LDH	122	U/L	<Tumor marker>			FT3	2.07	pg/mL
U-K	30	mEq/L	γ-GTP	48	U/L	CEA	1.5	ng/mL	FT4	0.86	ng/dL
U-Ca	27.6	mg/dL	ALP	407	U/L	CA19-9	10.7	U/mL	PTH-intact	75	pg/mL
U-IP	95.2	mg/dL	TP	6.7	g/dL	<Bone marker>			PTHrP	<1.1	pmol/L
FECa	0.98	%	Alb	4.0	g/dL	BAP	36.7	μg/L	1,25 (OH)2D	12.9	pg/mL
%TRP	83.4	%	Ch-E	291	U/L	NTx	31.5	nmolBCE/L	25 (OH)D	20	ng/mL
Tmp/GFR	1.59	mg/dL	UA	3.0	mg/dL	TRACP-5b	1330	mU/dL	FGF23	630	pg/mL

γ-GTP = gamma glutamyl transpeptidase, 1,25 (OH)_2_D = 1,25-dihydroxyvitamin D3, 25 (OH)D = 25-hydroxyvitamin D, ACTH = adrenocorticotropic hormone, Alb = albumin, ALP = alkaline phosphatase, ALT = alanine aminotransferase, AST = aspartate aminotransferase, BAP = bone alkaline phosphatase, BUN = blood urea nitrogen, CA19-9 = carbohydrate antigen 19-9, CBC = complete blood count, CEA = carcinoembryonic antigen, Ch-E = cholinesterase, Cre = creatinine, CRP = C-reactive protein, eGFR = estimated glomerular filtration rate, FGF23 = fibroblast growth factor 23, FSH = follicle-stimulating hormone, FT3 = free triiodothyronine, FT4 = free thyroxine, GH = growth hormone, Hb = hemoglobin, Ht = hematocrit, IGF-1 = insulin-like growth factor 1, LDH = lactate dehydrogenase, LH = luteinizing hormone, NTx = type 1 collagen cross-linked N-terminal telopeptide, pH = power of hydrogen, Plt = platelet, PRL = prolactin, PTH-intact = intact parathyroid hormone, PTHrP = parathyroid hormone-related peptide, RBC = red blood cell, T-bil = total bilirubin, TmP/GFR = tubular maximum reabsorption of phosphate to glomerular filtration rate, TP = total protein, TRACP-5b = tartrate-resistant acid phosphatase 5b, TRP = tubular reabsorption of phosphate, TSH = thyroid-stimulating hormone, UA = uric acid, WBC = white blood cell.

Dual-energy X-ray analysis showed decreased bone density with T scores of −2.2SD and −2.1SD for the total lumbar spine and femoral neck, respectively. Magnetic resonance imaging short TI inversion recovery (MRI) for whole body including head and neck, demonstrated high signal intensity in the talar, sacral, bilateral clavicle, and L5 vertebral regions, indicating multiple pseudofractures. Comprehensive imaging studies including systemic computed tomography and ^111^In-pentetreotide scintigraphy failed to reveal a tumor despite the clinical suspicion of TIO. Systemic venous sampling was then performed, and revealed high serum FGF23 of 479.0 pg/mL in the left external iliac vein compared with the other veins examined (Table 2). A second venous sampling limited to the left lower limb demonstrated high serum FGF23 of 326.1 pg/mL in the distal posterior tibial vein compared with the proximal posterior tibial vein (Table 2). However, an additional imaging study focused on the left foot did not identify a tumor. Genetic screening for potentially responsible genes including *Phosphate-regulating gene with homologies to endopeptidases on the X chromosome*, *Dentin matrix protein 1*, and *FGF23* showed no variations in these genes.

**Table 2:** Serum FGF23 levels in systemic and local venous samplings.

< Systemic venous sampling>			<Second venous sampling >	
	Right	Left		
Internal jugular vein	432.8	392.8	External iliac vein	221.7
Subclavian vein	328.6	336.9	Deep femoral vein	209.5
Common iliac vein	379.7	414.2	Great saphenous vein	249.4
Internal iliac vein	362.5	339.7	Poplireal vien	234.0
External iliac vein	356.9	479.0	Posterior tibial vein (proximal)	282.9
			(distal)	326.1

^∗^Serum FGF23 is presented in pg/mL. The second venous sampling was limited to the left lower limb.

**Figure 1:**
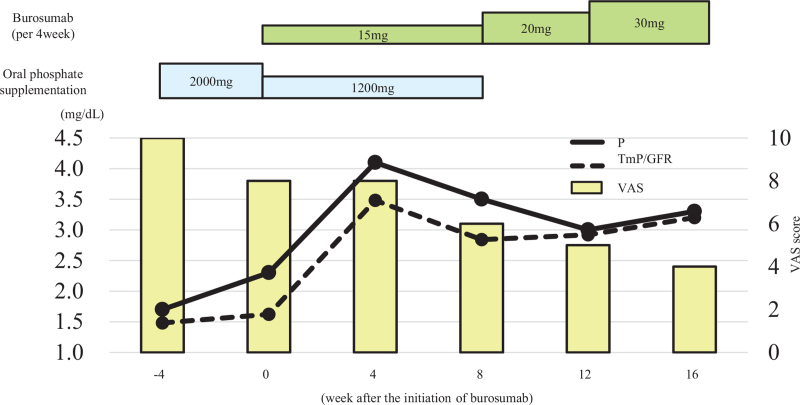
Changes in serum phosphate, renal tubular reabsorption of phosphate (TmP/GFR), and visual analog scale (VAS) score during the treatment course.

**Figure 2:**
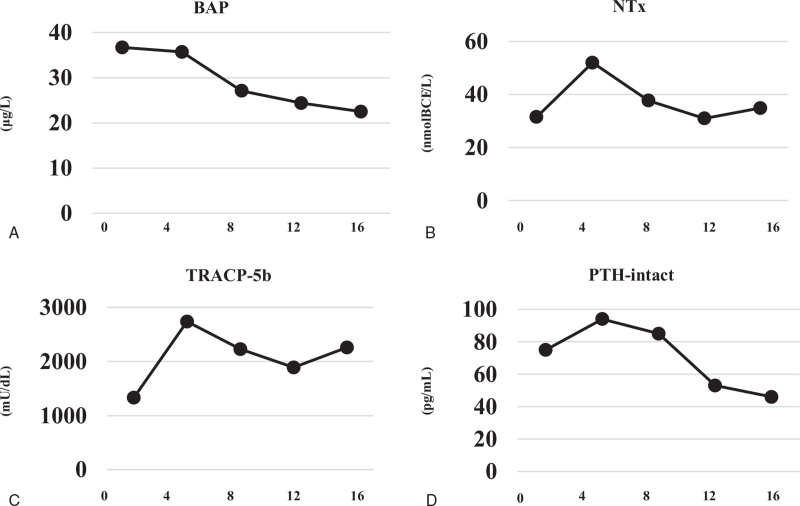
Changes in serum bone biomarkers. (A) Bone alkaline phosphatase (BAP). (B) Type 1 collagen cross-linked N-terminal telopeptide (NTx). (C) Tartrate-resistant acid phosphatase 5b (TRACP-5b). (D) Intact parathyroid hormone (PTH-intact).

**Figure 3:**
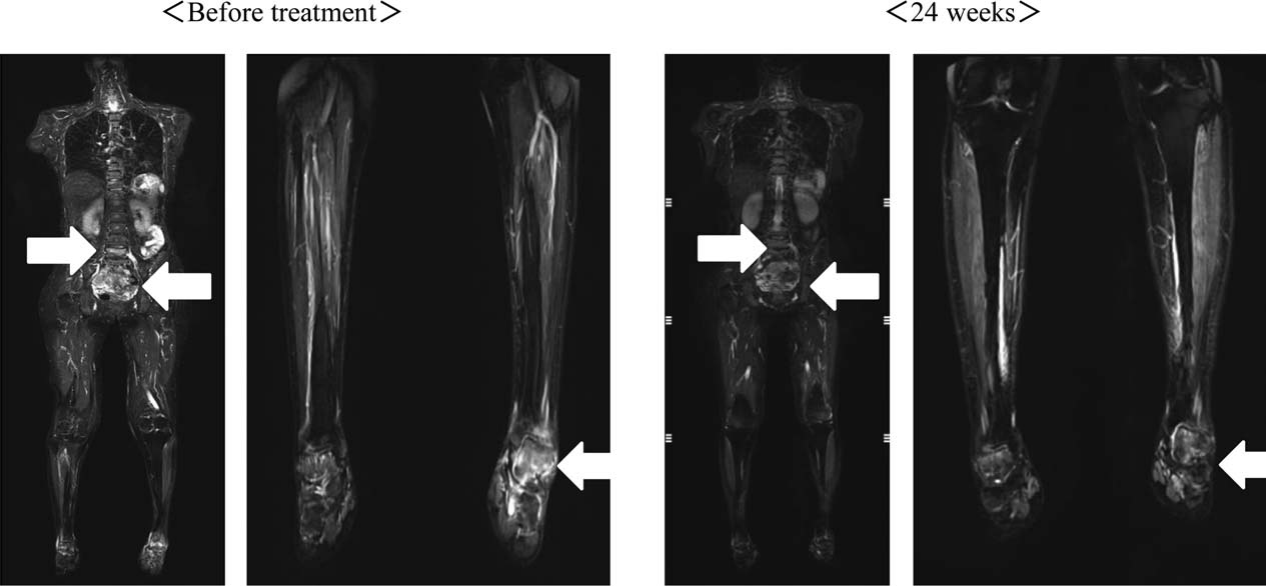
Short-tau inversion-recovery sequence magnetic resonance imaging of the whole body and lower legs. High signal intensity was observed in the talar, sacral, and L5 vertebral regions, indicating multiple pseudofractures that improved after treatment for 24 weeks (white arrows).

We decided to start phosphorus supplementation and subcutaneous anti-FGF23 antibody administration. The starting dose for burosumab was 15.0 mg (0.3 mg/kg) per month and gradually increased to 30.0 mg (Fig. 1). Phosphorus supplementation was stopped after the third administration of burosumab. Finally, serum phosphate and renal tubular reabsorption of phosphate turned normal at a dose of 30.0 mg without phosphate supplementation. During the 6-month course of burosumab administration, the bone resorption markers, TRACP-5b and NTx, initially increased but then gradually decreased after 8 weeks. Bone alkaline phosphatase as a bone formation marker showed a tendency to decrease slightly. The inappropriately elevated intact PTH given the high serum FGF23 gradually improved (Fig. 2). Physical pain evaluated by a visual analog scale (VAS) was 10/10 before administration of burosumab, and subsequently improved to 6/10 and 4/10 at 2 and 6 months, respectively (Fig. 1). Bone MRI revealed improvements in the pseudofractures in the talar, sacral, and L5 vertebral regions, excluding bilateral clavicle (Fig. 3). She was able to stand by herself at 6 months after initiation of burosumab treatment with no adverse events, although she had to use a wheelchair at the time of the consultation.

## 3 Discussion and conclusions

To the best of our knowledge, this is the first post-marketing report to demonstrate favorable effects, including improvements in subjective pain and pseudofractures, for burosumab administration in a case of TIO with undetectable tumor. Tumor localization can be challenging in TIO, and treatment of TIO with unknown tumor location is extremely difficult.^[11]^ Previously, therapeutic effects of burosumab for TIO were confirmed, but the reports were limited to detectable tumors including unresectable or recurrent cases. Although a few clinical trials have been performed on TIO cases with undetectable tumors, this is the report firstly would suggest therapeutic effects for TIO with undetectable tumor in a practical setting.

Day et al reported therapeutic effects of burosumab for an unresectable case of TIO.^[7]^ Although 2 tumors were identified, surgical resection was not performed due to their locations and duality. Medical treatment was therefore indicated as an alternative, and burosumab was administered. The administration dramatically improved serum phosphate walking ability and pain.

Miyaoka et al compared burosumab with conventional therapy for a recurrent TIO case.^[8]^ Despite repeated surgery for multiple recurrences, improvements in symptoms including bone pain and resection were not completed. Oral phosphate supplementation did not prevent decreases in bone mineral density and the patient was switched to burosumab after a long follow-up period of >10 years. After administration of burosumab, serum phosphate immediately normalized and bone density improved during 6 months.

A retrospective review of 144 cases of TIO in China described difficulty in reaching a diagnosis.^[11]^ In the study, the initial misdiagnosis rate was 95.1% and the mean time to TIO diagnosis was 2.9 ± 2.3 years from onset of symptoms. The delay of diagnosis may be related with the following, the symptoms of TIO are often non-specific, the tumors are typically very small in size and slow-growing. Moreover, the location of tumor varies rendering their localization complex.^[4]^ Although population-based epidemiological studies have not been conducted worldwide, the recognition of this rare disease is relatively poor and there may be more undiagnosed patients.

The observed changes in bone markers are another feature of the present case report. Burosumab normalized serum phosphate and the patient did not ultimately require oral phosphate supplementation. In a phase 2 open-label trial for TIO, serum phosphate was normalized by burosumab administration, with initial increases in bone markers in the early phase after administration followed by gradual decreases.^[9]^ It was suggested that the initial increase in bone alkaline phosphatase may reflect a compensatory mechanism for the previous inappropriate bone turnover in osteomalacia and the fact that receptor activator of nuclear factor-kappa B ligand is inhibited by long-term illness in TIO, suppressing bone resorption.^[12]^ However, bone resorption is activated by initiation of burosumab, triggering temporary increases in NTx and TRACP-5b. Activated bone metabolism is ameliorated by burosumab administration, resulting in gradual normalization of bone markers. During this period, calcium required used for bone formation, thus PTH is also inappropriately elevated in the early phase. Our case followed the same course as the previous report regarding changes in bone markers. Pseudofractures revealed by whole-body bone scintigraphy in a trial showed partial healing in 23.8% of cases.^[10]^ Although there were differences in devices, we also found partial healing of pseudofractures by short TI inversion recovery MRI. The distance for the 6-min walk test was significantly extended in the trial, and in our case, improvement in pain evaluated by the VAS and let the patient stand by herself.

We suspected TIO from adult-onset disease with no medication from the laboratory findings including hypophosphatemia and high serum FGF23, but could not localize the tumor. In addition to venous sampling, a stepwise method combining functional and anatomical imaging is recommended for tumor localization in TIO.^[13,14]^ A study involving selected venous samplings reported that FGF23 concentration ratio (between venous drainage of the tumor bed and the general circulation) >1.6 can be considered a diagnostic cut-point, with sensitivity of 87.0% and specificity of 71.0%.^[5,14]^ Nevertheless, the highest serum FGF23 was observed in the left external iliac vein in our case, with a concentration ratio of <1.6. In our case of FGF23-related hypophosphatemia, we excluded adult-onset autosomal dominant hypophosphatemic rickets by negative genetic screening, and subsequently initiated administration of burosumab for TIO with undetectable tumor.

^111^In-pentetreotide scintigraphy is commonly used worldwide for functional imaging in evaluating TIO.^[15]^ Although its specificity is high at 80.0%, the sensitivity is inadequate at 36.3% and there remains a possibility of inappropriate screening for the absence of identified tumor location.^[16]^ Meanwhile, ^68^Ga-conjugated somatostatin peptide analogues, such as ^68^Ga-DOTATATE PET/CT, can be useful for identifying phosphaturic mesenchymal tumors, with sensitivity of 54.5% and specificity of 85.7%.^[16]^ Because only limited facilities are able to perform ^68^Ga-DOTATATE PET/CT in Japan, unfortunately we were unable to use this modality.

As a limitation, we could not confirm improvement of mineralization by bone biopsy examination. In conclusion, we demonstrated favorable effects of burosumab on TIO with undetectable tumor, revealing improvements in both subjective pain by VAS evaluation and pseudofractures by MRI. Moreover, we observed normalization of bone biomarkers after burosumab administration.

## Acknowledgments

The authors thank Alison Sherwin, PhD, from Edanz Group (https://en-author-services.edanz.com/ac) for editing a draft of this manuscript.

## Author contributions

**Conceptualization:** Yuki Oe.

**Data curation:** Yuki Oe, Hiraku Kameda.

**Investigation:** Yuki Oe, Keita Sakamoto, Takeshi Soyama, Koji Iwasaki.

**Supervision:** Hiraku Kameda, Hiroshi Nomoto, Kyu Yong Cho, Akinobu Nakamura, Hideaki Miyoshi, Tatsuya Atsumi.

**Writing – original draft:** Yuki Oe.

**Writing – review & editing:** Hiraku Kameda, Hiroshi Nomoto, Keita Sakamoto, Takeshi Soyama, Kyu Yong Cho, Akinobu Nakamura, Koji Iwasaki, Daisuke Abo, Kohsuke Kudo, Hideaki Miyoshi, Tatsuya Atsumi.
